# Fertilization decreases the effect of ammonium nitrogen on microorganisms in Chinese *Carex tibetikobresia* meadows during rest-grazing

**DOI:** 10.3389/fmicb.2025.1608011

**Published:** 2025-06-18

**Authors:** Xuanbo Zhou, Xiaoli Wang, Yushou Ma, Yanlong Wang, Yuan Ma, Lele Xie

**Affiliations:** State Key Laboratory of Plateau Ecology and Agriculture, Academy of Animal Husbandry and Veterinary Sciences, Qinghai University, Xining, China

**Keywords:** fertilization, rest-grazing, soil microbial community, *Carex tibetikobresia* meadow, nutrient limitation

## Abstract

**Introduction:**

On degraded grasslands, rest-grazing and fertilization measures have been widely applied. In alpine grasslands, numerous studies have examined the impact of rest-grazing and fertilizer application on microbial communities. However, the impact of these measures on the microbial community in *Carex tibetikobresia* meadows remains largely understudied. Furthermore, the relationship between aboveground vegetation and soil components under these treatments warrants further investigation.

**Methods:**

We conducted a field control experiment in Dawu Town, Maqin County, China, during the winter–spring pasture regreen-up period. The primary treatment consisted of five rest-grazing durations, while the secondary treatment involved nitrogen addition.

**Results and discussion:**

The results indicated that, under rest-grazing treatment, the levels of soil nitrogen can improve and ammonium nitrogen (NH₄^+^-N) was the primary environmental factor affecting microbial biomass. It showed a significantly negatively correlated with bacteria and gram-negative bacteria (G^−^), but a positive correlation with the ratio of gram-positive bacteria to gram-negative bacteria (G^+^:G^−^). Furthermore, without fertilization treatment, the ratio of fungi to bacteria (F:B) and G^+^:G^−^ reached a maximum at rest-grazing for 30 days. In contrast, under fertilization treatment, microbial biomass carbon (MBC) became the dominant environmental factor affecting microbial biomass. It was negatively correlated with G^−^, but positively correlated with the ratio of F:B and G^+^:G^−^. Rest-grazing increases soil inorganic nitrogen and promotes actinomycetes growth, providing a viable strategy for restoring inorganic nitrogen levels in degraded grasslands. On the other hand, fertilization reduced the biomass of total phospholipid fatty acids (PLFAs) and all PLFAs groups. Consequently, the recommendation is that fertilization measures should not be utilized on this grassland and that a 30-day rest-grazing durations is preferable. Additionally, we observed inconsistent responses of microbial communities in the *Carex tibetikobresia* meadow and alpine meadows to rest-grazing and fertilization. These findings offer valuable insights into how fertilization modifies microbial responses to rest-grazing, providing important guidelines for the management of degraded *Carex tibetikobresia* meadows.

## 1 Introduction

The *Carex tibetikobresia* meadow is a significant vegetation type in the three-river source region, serving as an excellent grazing area and an important site for water conservation (Yu et al., 2010). Recently, this meadow ecosystem has experienced significant degradation due to climate warming and unsustainable anthropogenic disturbances, particularly intensive overgrazing, resulting in substantial declines in vegetation productivity and accelerated soil erosion rates (Li et al., 2019). Grassland degradation reduces vegetation cover and diversity, reducing soil nitrogen levels (Teng et al., 2020). These changes profoundly impact soil microbial biomass and community structure (Zhang et al., 2023). Soil nitrogen levels influence the composition of microbial communities and the capacity of microorganisms to acquire carbon (Yang A. et al., 2024). Therefore, studying soil nitrogen levels is crucial for grassland restoration (Yang S. et al., 2024). The health and function of grasslands are closely related to soil quality, and implementing better grazing management systems can support the sustainable development of the grassland ecosystem (Feng et al., 2024). However, the dearth of research on the *Carex tibetikobresia* meadows in this region is evident.

Soil microorganisms play a vital role in nutrient cycling and utilization, maintaining the function and stability of the grassland ecosystem, which is a crucial component of this environment (Bilen and Turan, 2022). Moreover, soil microorganisms influence plant growth (Zhang et al., 2019). This may be because rest-grazing can reduce vegetative root biomass while increasing soil fungal biomass (Li et al., 2022). The analysis of phospholipid fatty acids (PLFAs) is considered an accurate measure of viable biomass in soil microbial communities, making PLFAs measurements useful indicators for evaluating the effectiveness of vegetation restoration (Hu et al., 2020). Zhao et al. (2017) demonstrated that grazing significantly reduced total microbial, bacterial, and fungal communities, indicating that soil microbial community sizes were significantly influenced by grazing intensity. Consequently, microbial indicators serve as essential biomarkers for evaluating grassland restoration success. Nevertheless, there is a paucity of research on the effects of rest duration on microorganisms.

Rest-grazing is widely recognized as a nature-based restoration method for rehabilitating degraded grasslands throughout the three-river source region to promote the healthy development of grasslands (He et al., 2020). While most studies to date have focused on rest-grazing, rotational grazing, and fenced enclosures, the optimal duration of rest-grazing during the regreen-up period remains unclear (Venter et al., 2019). Fertilization is an effective management strategy for degraded grasslands, improving the stability of vegetation communities (Sheng et al., 2022). However, there are few reports on the combined effects of rest-grazing and fertilization in degraded grasslands (Zong et al., 2021). To address this gap, we conducted experiments on rest-grazing duration and fertilization in alpine meadows during the early growth stage and obtained relevant results (Zhou et al., 2023). Nevertheless, since *Carex tibetikobresia* meadows possess higher soil nutrient levels than alpine meadows, soil microorganisms may respond differently. In recent years, research on soil microorganisms has primarily focused on various restoration strategies, with limited studies addressing the effects of rest-grazing duration and fertilization on *Carex tibetikobresia* meadows in the three-river source region (Sadeghi et al., 2023). Considering these ecological impacts, the optimization of grazing regimes and fertilization protocols is essential to facilitate vegetation recovery and improve soil health in degraded grasslands.

To investigate how rest-grazing duration and fertilizer application treatments impact soil microbial communities in *Carex tibetikobresia* meadows, we formulated the following hypotheses: (1) Rest-grazing can improve the levels of soil nitrogen; (2) After applying nitrogen fertilizer, the ability of microorganisms to acquire carbon will be enhanced; (3) The responses of soil microorganisms in *Carex tibetikobresia* meadows and alpine meadows to rest-grazing and fertilizer application will differ. To test these hypotheses, we employed PLFAs analysis to evaluate the changes in biomass of each group of soil microorganisms. We identified the primary drivers’ factors influencing these variations under varying rest-grazing durations and fertilization treatments.

## 2 Materials and methods

### 2.1 Study area

This research was conducted in the winter–spring pasture of Dawu Town, Guoluo Tibetan Autonomous Prefecture, Qinghai Province, China (34°27′52″N, 100°13′19″E), at an altitude of 3,728 m. This area belongs to the alpine climate of the Qinghai-Tibet Plateau (Mao et al., 2021). The study area exhibits the climate with an average annual temperature of −3.9°C (summer: 9.7°C, winter: −12.6°C), annual precipitation ranging from 513.2 to 542.9 mm predominantly concentrated in June–September, and a permanent frost presence despite having approximately 156 frost-free days supporting vegetation growth (Li et al., 2020). The grassland type is classified as *Kobresia tibetica* swamp meadow, dominated by *Kobresia tibetica*, with coexisting species including *Blysmus sinocompressus*, *Carex moorcroftii*, *Kobresia pygmaea*, *Deschampsia caespitosa*, *Elymus nutans*, *Potentilla anserina*, and *Trollius pumilus* (Tian et al., 2021).

### 2.2 Experimental design

The primary utilization mode of the *Carex tibetikobresia* meadow is for the grazing of yaks, where herders have developed various seasonal grazing regimes, including winter–spring, summer, and autumn. In this study, we focused exclusively on the winter–spring grazing regime. Over the past several years, the experimental site has been grazing in winter–spring period. In order to reduce the spatial heterogeneity, the objective was to select grassland with the same stand conditions when choosing sample plots for the experiment. Grazing exclusion through mesh fencing has been implemented to mitigate grassland degradation in the *Carex tibetikobresia* meadow (Zhao et al., 2016b). In order to evaluate the effects of rest-grazing and fertilizer applicationon the *Carex tibetikobresia* meadow, we established a primary treatment of five rest-grazing durations and a secondary treatment of fertilization within each primary treatment. The five rest-grazing durations included: (1) rest-grazing for 20 days (20d), (2) rest-grazing for 30 days (30d), (3) rest-grazing for 40 days (40d), (4) rest-grazing for 50 days (50d), and (5) continuous grazing throughout the winter–spring period, serving as the control check group (CK). The grazing intensity in this study was based on the actual grazing intensity (moderate and light grazing) of the herders, which ranged from approximately 0.89 to 1.45 cattle per hectare.

### 2.3 Treatment applications

**Figure 1:**
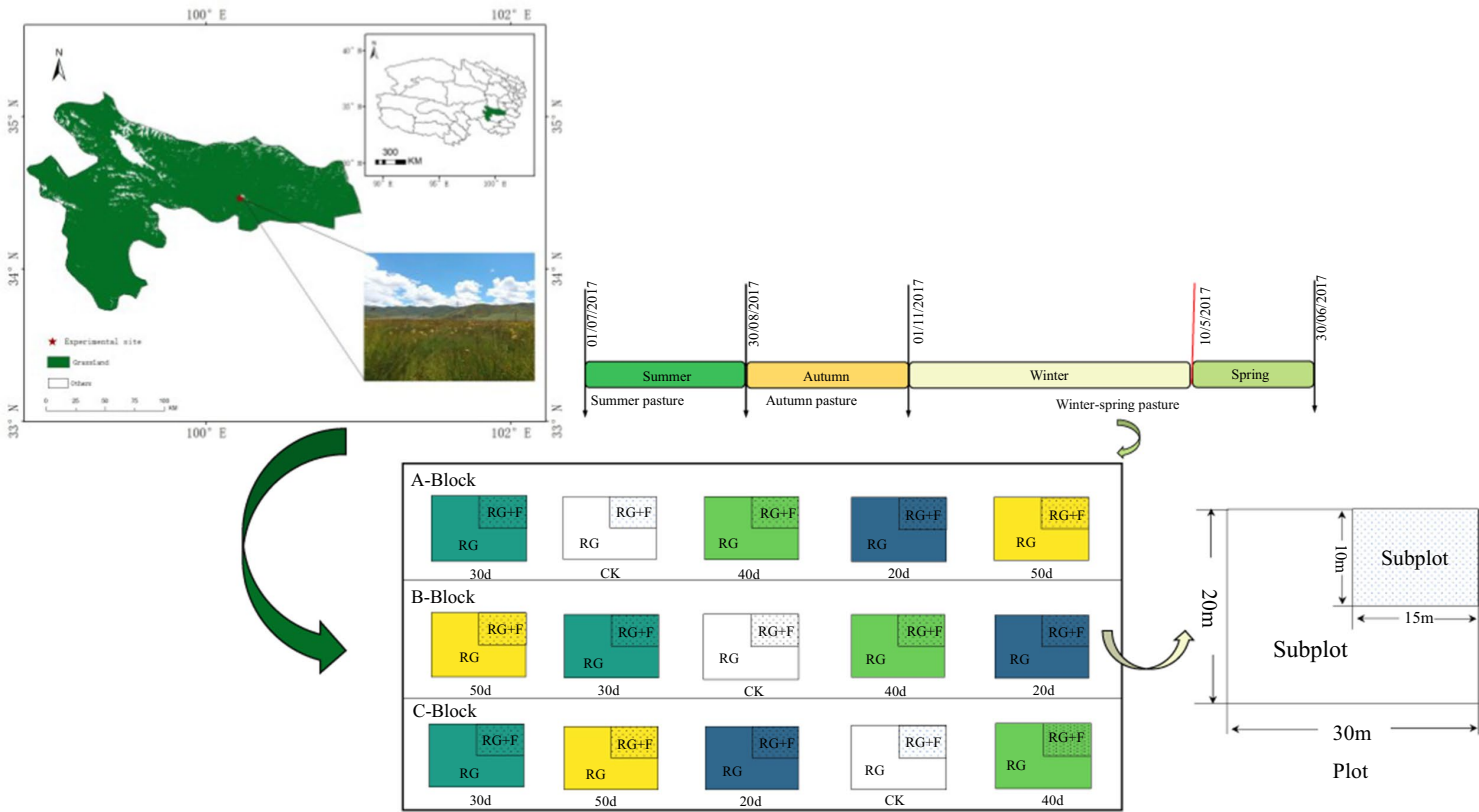
Layout of the field control experiment. Winter–spring pasture was grazing in winter and spring but rest-grazing in summer and autumn. A-Block, B-Block and C-Block: three independent replicate blocks; CK, control check group; 20d, rest-grazing from June10th to June 30th; 30d, rest-grazing from May 30th to June 30th; 40d, rest-grazing from May 20th to June 30th; 50d, rest-grazing May 10th to June 30th; RG, rest-grazing treatment; RG + F, rest-grazing with fertilization treatment; there were 2 subplots in each plot and there were 5 plots in each block.

On 20 June 2017, a one-time application of approximately 300 kg·ha^−1^ of nitrogen fertilizer (urea N content was 46%) was made, based on the findings of the study conducted on degraded grassland under consistent conditions (Zong et al., 2014). In the present study, three independent repetitive blocks (A, B, and C) were established as replicates for each treatment, with five plots (CK, 20d, 30d, 40d, and 50d) of 600 m^2^ (30 m × 20 m) randomly set up within each block. In each plot subjected to the rest-grazing treatments, we set up fertilization treatment as a subplot (15 m × 10 m), which is located in the northeast corner of each plot (Figure 1). A total of 15 experimental plots, with each plot divided into two subplots, resulting in a total of 30 subplots (3 replicate blocks × 5 plots × 2 subplots). Plant sampling involved clipping grass at ground level, sorting by species, and bagging the samples (Wu et al., 2023). Three plant quadrats were established within each subplot to minimize spatial heterogeneity, resulting in 90 plant samples (3 vegetation quadrats × 30 subplots). Soil sampling was conducted by obtaining three randomly distributed samples from each subplot, resulting in 90 individual specimens for laboratory processing. After measuring the plant and soil samples, data for each subplot were expressed as the mean of three samples.

### 2.4 Plant investigation and analysis

#### 2.4.1 Plant quadrat surveys

August 2017, we assessed the coverage, height, and aboveground biomass of all species in each vegetation quadrat. The calculation of the aboveground biomass (g·m^−2^) was based on the dry weight of the plant samples. The above-ground parts of the plants were collected, and subjected to a drying process at 65°C for a period of 48 h, following which they were analyzed.

#### 2.4.2 Characteristics analysis of vegetation community

The Shannon-Wiener index (H), Simpson index (D), and Pielou evenness index (J) were calculated using plant importance values (Pi):

Shannon-Weiner index (*H*) = −∑Pi * ln Pi.

Simpson index (*D*) = 1 − ∑Pi^2^.

Pielou index (*J*) = H/ln S.

Where Pi represents the important value of each species, and S represents the number of species in the plant quadrat.

### 2.5 Soil sampling and analysis

#### 2.5.1 Collection and storage of soil

Soil samples were collected and processed following a structured protocol. A soil sample is constituted by the combination of five soil cores extracted at a depth of 0 to 15 cm, using a soil auger with an inner diameter of 3.5 cm. Each sample was sieved through a 2 mm mesh and divided into three parts upon returning to the laboratory (Joshi and Garkoti, 2023). One portion of the fresh soil was stored at 4°C for microbial biomass analysis (carbon and nitrogen), inorganic nitrogen (NH₄^+^-N and NO_3_^−^-N), and available phosphorus (AP). The second portion was air-dried indoors to determine soil physicochemical properties (Zhao et al., 2018). The third portion was stored in a refrigerator at −80°C for PLFAs analysis.

#### 2.5.2 Determination of soil characteristics

Soil factors included soil moisture (SM), the potential of hydrogen (pH), soil organic carbon (SOC), total nitrogen (TN), ammonia nitrogen and nitrate nitrogen (NH₄^+^-N and NO_3_^−^-N), total phosphorus (TP), AP, total potassium (TK), and microbial biomass carbon and nitrogen (MBC, MBN). SM (%) was determined by the drying method. Soil pH was determined in a 1:5 soil-to-water suspension using a pH meter (METTER TOLEDO) (Su et al., 2005). SOC (g·kg^−1^) was quantified using potassium dichromate (K₂Cr₂O₇) oxidation followed by ferrous sulfate (FeSO₄) titration. TN (g·kg^−1^) was determined by means of the Kjeldahl method. NH₄^+^-N (g·kg^−1^) and NO_3_^−^-N (g·kg^−1^) were determined by potassium chloride leaching method. TP (g·kg^−1^) was determined by the molybdenum antimony colorimetric method and AP (g·kg^−1^) by the sodium bicarbonate extraction-molybdenum-antimony anti-colorimetric method. TK was assessed using a flame photometer (Huang et al., 2014). Finally, MBC (g·kg^−1^) and MBN (g·kg^−1^) were determined using chloroform fumigation extraction (Brookes et al., 1985).

### 2.6 Determination of phospholipid fatty acids

Microbial PLFAs were analyzed based on the method described by Bossio and Scow to elucidate the effects of rest-grazing and fertilization on the structure of the soil microbial community in the *Carex tibetikobresia* meadow (Bossio and Scow, 1998). Eight grams of lyophilised soil sample were used for the purposes of this analysis. The specific steps of the experiment were adapted from Zhou et al. (2023). The PLFA analysis method is based on phospholipids, which are an important component of microbial cell membranes (White et al., 1979). The classification is based on the observation that gram-positive bacteria (G^+^) have a thick peptidoglycan layer dominated by branched-chain saturated fatty acids, whereas gram-negative bacteria (G^−^) have thin cell membranes dominated by monounsaturated fatty acids (Ji et al., 2020). Fungal cell membranes have been found to contain complex lipids, such as ergocalciferol, and are dominated by long-chain polyunsaturated fatty acids (Ji et al., 2020). The fatty acid synthesis pathway in Actinomycetes (Act) adds a methyl group to the fatty acids’10th carbon (Ji et al., 2020). The following classification has been proposed for this. Arbuscular mycorrhizal fungi (AMF) were identified by the presence of the fatty acid 16:1ω5c (Bossio and Scow, 1998). Act were represented by the fatty acids 10Me-16:0, 10Me-17:0, and 10Me-18:0 (Kroppenstedt, 1985). Fungi (F) were characterized by 18:2ω6, 9c and 18:1ω9c (Frostegard et al., 1993). The values of i14:0, i15:0, a15:0, i16:0, a17:0, and i17:0 were used to represent G^+^, while 16:1ω7c, cy-17:0, 18:1ω5c, 18:1ω7c, and cy-19:0 were used to represent G^−^ (Zhang et al., 2022). Bacteria (B) were characterized by the following fatty acids: 14:0, i14:0, 15:0, i15:0, a15:0, 16:0, i16:0, 16:1ω7c, 17:0, a17:0, i17:0, cy-17:0, 18:0, 18:1ω5c, 18:1ω7c, and cy-19:0 (White et al., 1979). Additionally, the ratio of fungi to bacteria (F:B) and the ratio of gram-positive bacteria to gram-negative bacteria (G^+^:G^−^) were calculated (Sadeghi et al., 2023). Bacterial stress indicators (cy:pre) were also calculated as the ratio of (cy-17:0 + cy-19:0) to (16:1ω7c + 18:1ω7c) (Fierer et al., 2003). The total PLFAs was calculated by summing the PLFAs in each group.

### 2.7 Statistical analysis

Initially, raw data were summarized and collated using MS Excel and data were tested and analyzed using Statistical Package for Social Sciences 25.0. Comparisons between the five rest-grazing durations were made using one-way ANOVA and Least Significant Difference Tests, and independent samples *t*-tests were used to test for differences between fertilization and non-fertilization treatments. Differences between microbial communities in the rest-grazing were tested by Nonmetric multidimensional scaling (NMDS), and linear regression was used to test the relationship between microbial community composition and plant and soil properties. Finally, redundancy analysis (RDA) was used to screen the effects of environmental factors on microbial communities. Scatter charts and histograms were generated using Origin 2021 and RDA analysis was performed using CANOCO 5.0. In all tests, significance levels were set at *p* < 0.05 or *p* < 0.01.

## 3 Results

### 3.1 Responses of plant and soil properties to rest-grazing duration and fertilization

**Figure 2:**
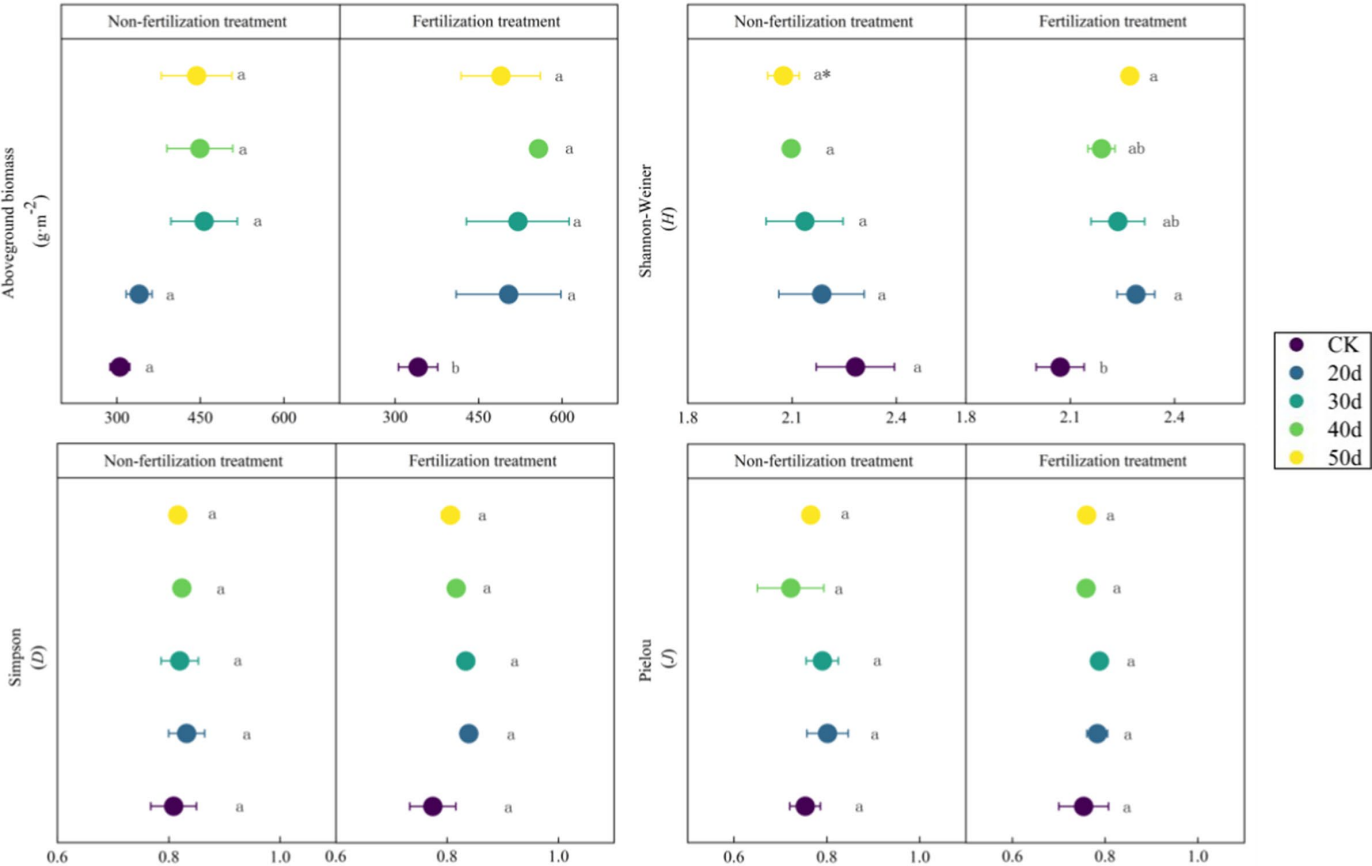
The biomass of aboveground vegetation and plant communities’ diversity index. These scatter diagrams show the biomass of aboveground vegetation and plant communities’ diversity index (Shannon-Weiner, Simpson, Pielou) under rest-grazing and fertilization treatments. Different lowercase letters in the same picture indicate significant differences between different days of rest-grazing under the same treatment; * means significant difference between non- fertilization and fertilization under the same number of rest-grazing days, *, *p* < 0.05. CK, control check group; 20d, rest-grazing from June10th to June 30th; 30d, rest-grazing from May 30th to June 30th; 40d, rest-grazing from May 20th to June 30th; 50d, rest-grazing May 10th to June 30th, (*n* = 3).

Implementation of rest-grazing and fertilization treatments induced significant modifications in both plant community traits and soil physicochemical properties (Figure 2; Table 1). Rest-grazing alone did not significantly impact the aboveground vegetation characteristics; however, fertilization under rest-grazing conditions significantly modified the aboveground biomass and Shannon-Wiener index (Figure 2). AP and SOC were significantly influenced by rest-grazing measures (*p* = 0.003, *p* = 0.015) (Table 1). In contrast, fertilization significantly affected soil C:N ratio TP, TN, and the (*p* = 0.001, *p* = 0.034 and *p* = 0.001) (Table 1). Compared to non-fertilized conditions, fertilization significantly increased SOC, TN, and TK but decreased the soil C:N ratio, AP, and NO_3_^−^-N. When comparing non-fertilized and fertilized conditions, fertilization increased SOC, TN, and TK while decreasing the soil C:N ratio, AP and NO_3_^−^-N (Supplementary Table S1).

**Table 1:** Variability analysis of soil physicochemical properties.

Soil physicochemical properties	Non-fertilization		Fertilization	
	*F*	*p*	*F*	*p*
SM	1.790	0.207	0.671	0.627
pH	2.925	0.077	1.553	0.260
NH₄^+^-N	2.310	0.129	0.406	0.801
NO_3_^−^-N	1.265	0.346	0.289	0.878
TP	1.508	0.272	3.996	0.034*
AP	8.274	0.003**	1.043	0.433
TK	2.086	0.158	2.243	0.137
SOC	3.958	0.035*	0.878	0.510
TN	2.929	0.077	35.966	0.001**
Soil C:N	5.267	0.015*	34.926	0.001**

* means significant difference between different days of rest-grazing under the same treatment, *, *p* < 0.05, **, *p* < 0.01. SM, Soil moisture; pH, potential of hydrogen; NH₄^+^-N, ammonium nitrogen; NO_3_^−^-N, nitrate nitrogen; TP, total phosphorus; AP, available phosphorus; TK, total potassium; SOC, soil organic carbon; TN, total nitrogen; soil C:N, soil carbon-nitrogen ratio; (*n* = 3).

### 3.2 Responses of microbial community composition to rest-grazing duration and fertilization

**Figure 3:**
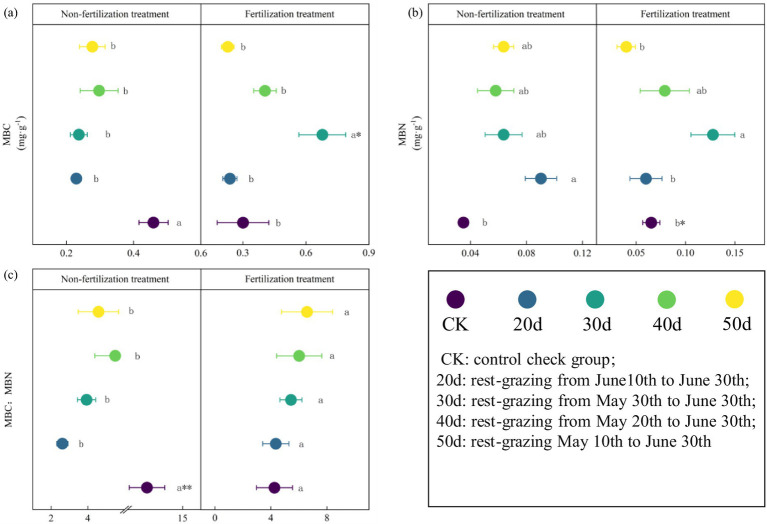
The biomass of soil microbial biomass carbon (MBC), nitrogen(N) and the ratio of MBC: MBN. These scatter diagrams show the biomass of soil microbial biomass carbon (MBC), soil microbial biomass nitrogen (MBN) and the ratio of MBC: MBN under rest-grazing and fertilization treatments. **(a)** The biomass of soil microbial biomass carbon; **(b)** the biomass of soil microbial biomass nitrogen; **(c)** the ratio of MBC: MBN. Different lowercase letters in the same picture indicate significant differences between different days of rest-grazing under the same treatment; * means significant difference between non- fertilization and fertilization under the same number of rest-grazing days, *, *p* < 0.05. CK: control check group; 20d: rest-grazing from June10th to June 30th; 30d: rest-grazing from May 30th to June 30th; 40d: rest-grazing from May 20th to June 30th; 50d: rest-grazing May 10th to June 30th, (*n* = 3).

**Figure 4:**
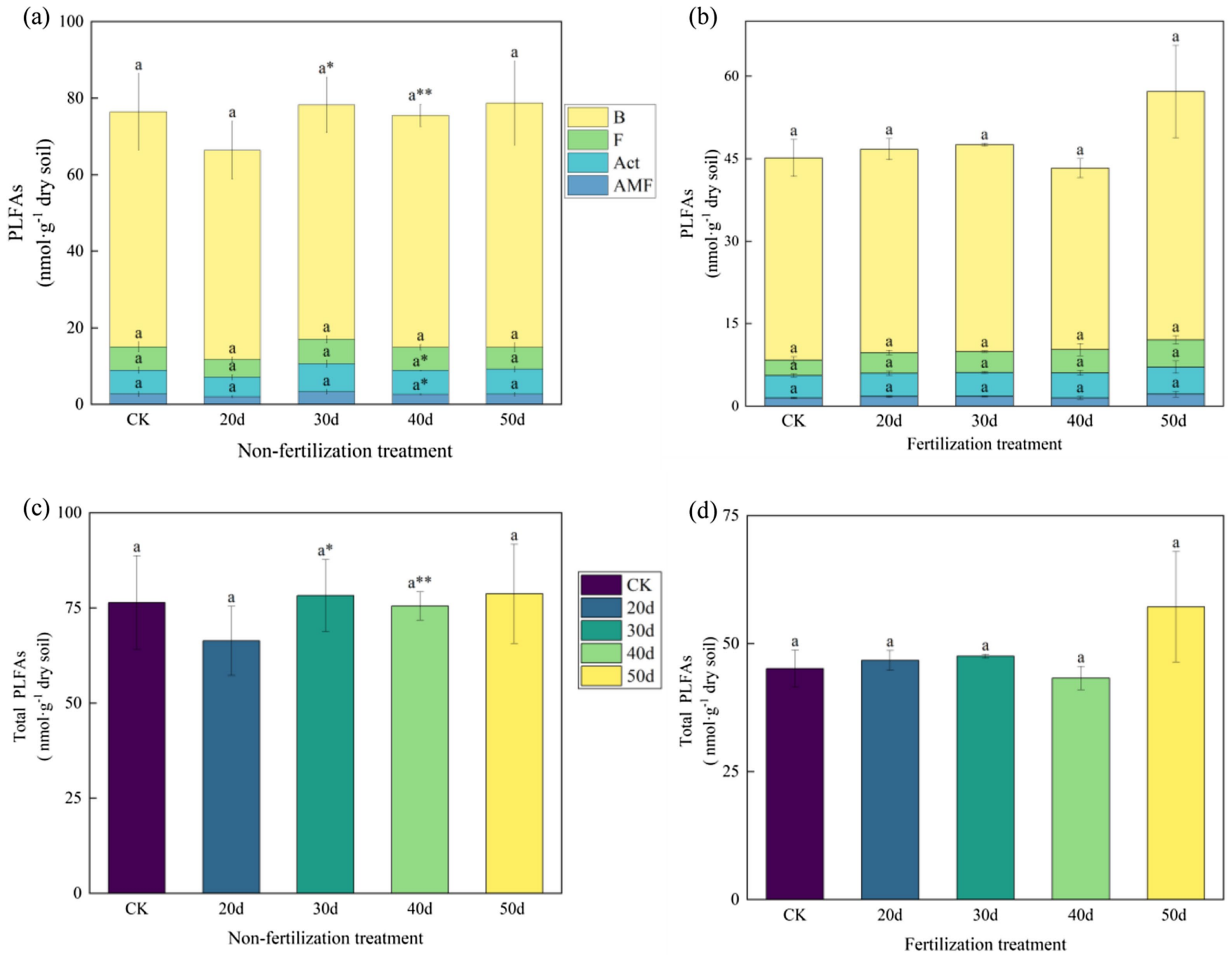
The content of phospholipid fatty acids in soil microorganisms. These histograms show the content of phospholipid fatty acids in soil microorganisms under rest-grazing and fertilization. **(a)** Contents of PLFAs groups under non-fertilization treatment; **(b)** contents of PLFAs groups under fertilization treatment; **(c)** contents of total PLFAs under non-fertilization treatment; **(d)** contents of total PLFAs under fertilization treatment. Different lowercase letters in the same picture indicate significant differences between different days of rest-grazing under the same treatment; * means significant difference between non- fertilization and fertilization under the same number of rest-grazing days, *, *p* < 0.05, **, *p* < 0.01. PLFAs: phospholipid fatty acids; B: total bacteria PLFAs; F: fungi PLFAs; Act: actinomyces PLFAs; AMF: arbuscular mycorrhizal fungi PLFAs; Total PLFAs: the sum of the PLFAs of each group of microorganisms. CK: control check group; 20d: rest-grazing from June10th to June 30th; 30d: rest-grazing from May 30th to June 30th; 40d: rest-grazing from May 20th to June 30th; 50d: rest-grazing May 10th to June 30th, (*n* = 3).

The soil MBC, MBN, and the MBC:MBN ratio were altered following rest-grazing and fertilization (Figure 3). Without fertilization, there were significant differences between the CK and rest-grazing groups for three metrics: the MBC:MBN ratio, MBC and MBN. At this time, from 20d to 50d of rest-grazing, MBC showed an inverted V-shaped change and reached its highest point at 40d, while MBN showed a V-shaped change and reached its lowest point at 40d (Figure 3). However, with fertilization, from 20d to 50d of rest-grazing, MBC and MBN showed an inverted V-shaped variation, reaching a maximum on 30d, which was significantly higher than in the other groups (Figure 3). Under both non-fertilizer and fertilizer treatments, maximum PLFAs were observed after 50d. Notably, fertilization reduced the biomass of total PLFAs and all PLFAs groups. After 40d, AMF, Bacteria and Act, values of non-fertilization treatment were significantly higher than those of the fertilization treatment (*p* = 0.018, *p* = 0.001 and *p* = 0.019) (Figure 4). Without fertilization, from 20d to 50d of rest-grazing, G^+^ showed an inverted V-shaped variation, reaching a maximum on 30d, while G^−^ showed a linear upward trend, reaching its maximum value after 50 days (Figure 4). However, with fertilization, from 20d to 50d of rest-grazing, G + showed an increasing and then decreasing trend, reaching a minimum at 40d. G- showed a V-shaped trend, also reaching a minimum at 40 d (Figure 4).

**Figure 5:**
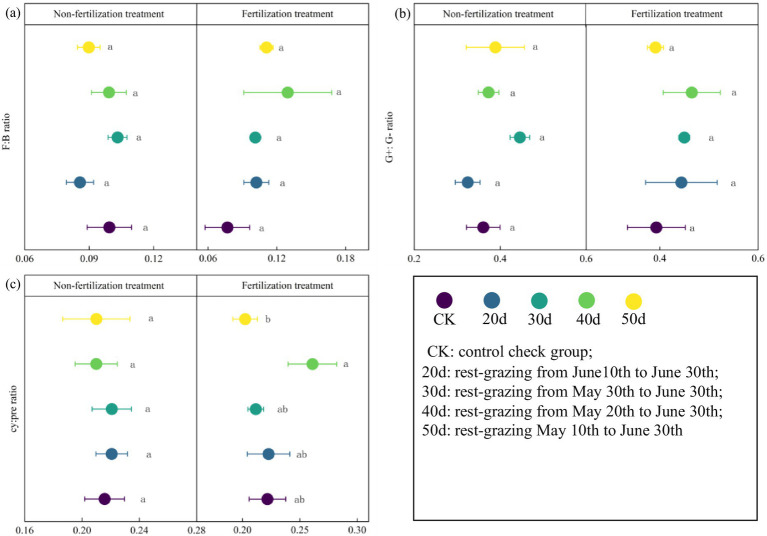
The stress indicators of phospholipid fatty acids in soil microorganisms. These scatter diagrams show the stress indicators of phospholipid fatty acids in soil microorganisms under rest-grazing and fertilization treatments. **(a)** The ratio of fungi to bacteria PLFAs; **(b)** the ratio of gram-positive to gram-negative bacteria PLFAs; **(c)** the ratio of (cy-17:0 + cy-19:0) to (16:1 ω7c + 18:1ω7c). Different lowercase letters in the same picture indicate significant differences between different days of rest-grazing under the same treatment. F: B ratio: the ratio of fungi to bacteria PLFAs; G^+^:G^−^ ratio: the ratio of gram-positive to gram-negative bacteria PLFAs; cy: pre ratio: the ratio of (cy-17:0 + cy-19:0) to (16:1 ω7c + 18:1ω7c). CK: control check group; 20d: rest-grazing from June10th to June 30th; 30d: rest-grazing from May 30th to June 30th; 40d: rest-grazing from May 20th to June 30th; 50d: rest-grazing May 10th to June 30th, (*n* = 3).

**Figure 6:**
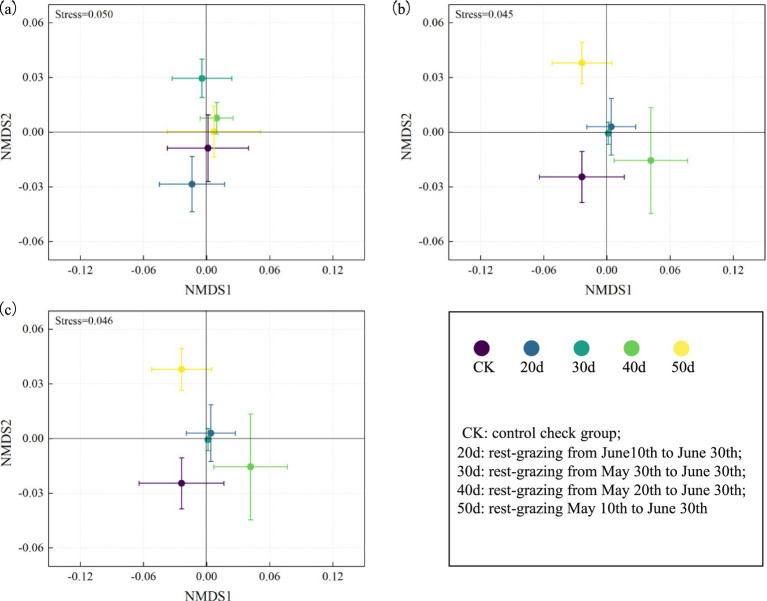
The microbial community compositions were analyzed by Nonmetric multidimensional scaling. These scatter diagrams show the microbial community compositions were analyzed by Nonmetric multidimensional scaling under rest-grazing and fertilization treatments. **(a)** Non-fertilization treatment; **(b)** fertilization treatment; **(c)** combined treatment of fertilization and non-fertilization. CK, control check group; 20d, rest-grazing from June10th to June 30th; 30d, rest-grazing from May 30th to June 30th; 40d, rest-grazing from May 20th to June 30th; 50d, rest-grazing May 10th to June 30th, (*n* = 3).

Furthermore, under rest-grazing measures, the mean of F:B ratios, G^+^:G^−^, and cy:pre ratios were highest after 30d. In contrast, with fertilization, these indicators peaked after 40d (Figure 5). NMDS results indicated significant differences in microbial community compositions across different rest-grazing durations. Specifically, under rest-grazing, NMDS2 was significantly higher at 30d than at 20d (*p* = 0.019) (Figure 6a). When considering fertilization under rest-grazing measures, NMDS2 was significantly higher at 50d than at CK (*p* = 0.024) (Figure 6b). A comparative analysis between non-fertilization and fertilization treatments revealed significantly greater differentiation in microbial community composition following fertilization (Figure 6c).

### 3.3 Soil and vegetation drivers of microbial community composition

**Figure 7:**
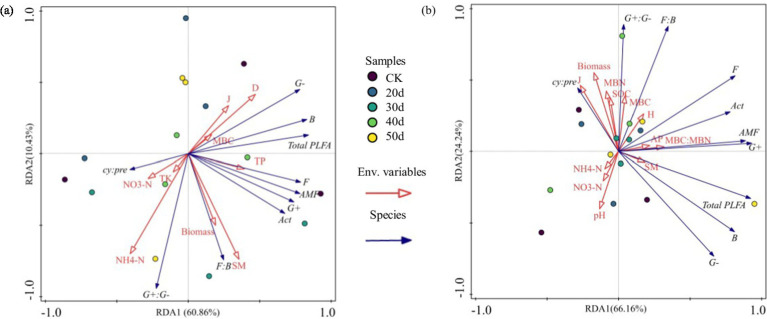
Redundancy analysis of soil physicochemical properties and environmental factors. **(a)** No fertilization treatment; **(b)** fertilization treatment; CK, control check group; 20d, rest-grazing from June10th to June 30th; 30d, rest-grazing from May 30th to June 30th; 40d, rest-grazing from May 20th to June 30th; 50d, rest-grazing May 10th to June 30th; Env. Variables, plant and soil properties namely environmental variables; Species, all PLFAs groups namely species variables; Act, actinomyces PLFAs; AMF, arbuscular mycorrhizal fungi PLFAs; B, total bacterial PLFAs; cy:pre, the ratio of (cy-17:0 + cy-19:0) to (16:1 ω7c + 18:1ω7c); F, fungal PLFAs; F:B, the ratio of fungal to bacterial PLFAs; G^−^, gram-negative bacterial PLFAs; G^+^, gram-positive bacterial PLFAs; G^+^:G^−^, the ratio of gram-positive to gram-negative bacterial PLFAs; Total PLFAs, the sum of the PLFAs of each group of microorganisms; D, Simpson index; SM, soil moisture; NH₄^+^-N, ammonium nitrogen; TP, total phosphorus; J, Pielou index; TK, total potassium; NO_3_^—^N, nitrate nitrogen; MBC, soil microbial biomass carbon; Biomass, the biomass of aboveground vegetation; H, Shannon-Weiner index; MBC:MBN, the ratio of soil microbial biomass carbon to soil microbial biomass nitrogen; AP, available phosphorus; pH, potential of hydrogen; MBN, soil microbial biomass nitrogen; SOC, soil organic carbon; (*n* = 3).

**Figure 8:**
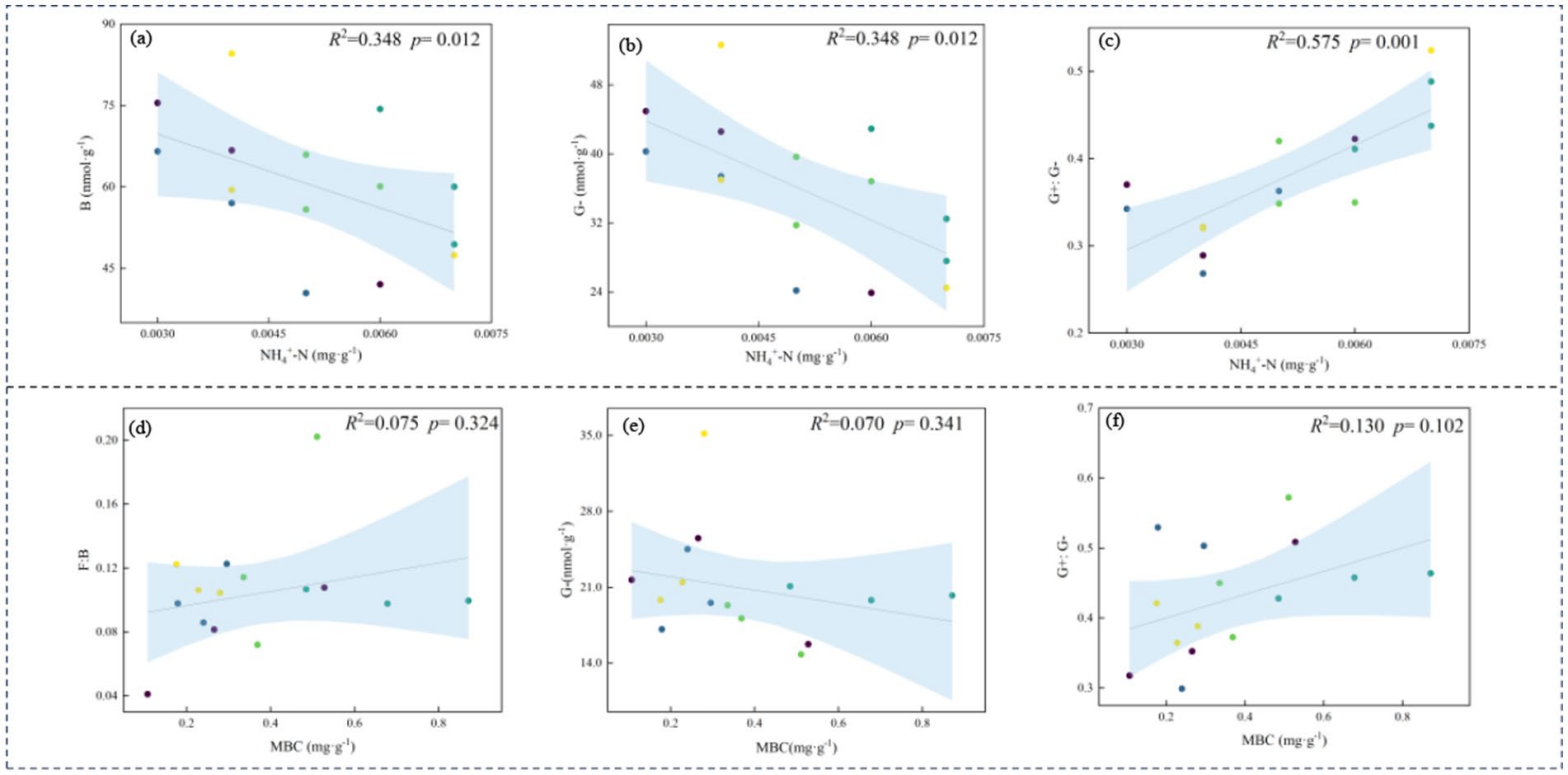
Relationships between the soil physicochemical properties and microbial properties. No fertilization treatment **(a–c)**; fertilization treatment **(d–f)**; NH₄^+^-N, ammonium nitrogen; MBC, soil microbial biomass carbon; B, total bacteria PLFAs; G^−^, gram-negative bacteria PLFAs; G^+^:G^−^, the ratio of gram-positive to gram-negative bacteria PLFAs; F:B, the ratio of fungi to bacteria PLFAs, (*n* = 3).

Both edaphic properties and vegetative characteristics collectively drive structural reorganizations in microbial communities (Figure 7). RDA indicated that, under rest-grazing measures, the microbial community was primarily influenced by NH₄^+^-N, Simpson index (D), and SM. After fertilization, MBC, MBN, and SOC mainly influenced the microbial community. Specifically, under rest-grazing conditions, NH₄^+^-N accounted for 22.80% of the variation (*p* = 0.042) (Supplementary Table S2); B and G^−^ decreased with increasing NH₄^+^-N, while G^+^:G^−^ increased (Figures 8a–c). Following fertilization, MBC explained 18.10% of the variation (*p* = 0.028) (Supplementary Table S2), with F:B and G^+^:G^−^ increasing alongside MBC, whereas G^−^ decreased (Figures 8d–f).

## 4 Discussion

### 4.1 Fertilization will change the effects of rest-grazing on plant and soil properties, and microorganisms

Rest-grazing and fertilization measures can benefit degraded grasslands and alter soil microbial communities (Clegg, 2006). This study uniquely examines how rest-grazing duration and fertilization regimes collectively structure soil microbial communities in the three-river source region. Our findings offer implementable solutions for rehabilitating degraded *Carex tibetikobresia* meadows and optimizing rotational grazing systems in fragile pastures.

Recent studies have shown that appropriate grazing pressure and nitrogen fertilizer application effectively enhance grassland productivity and maintain soil microbial community stability (Qi et al., 2023). According to results from NMDS, fertilization enhances the effects of rest-grazing duration on microbial communities (Figure 6). This may occur because fertilization disrupts the balance of the soil–plant system, thereby affecting the composition and abundance of microbial communities (Coonan et al., 2020). Additionally, we found that the fertilization treatment decreased the biomass of all PLFAs groups and total PLFAs (Figure 4). This finding is consistent with previous research indicating an average biomass decrease of 15% for the entire microbial community under nitrogen fertilization (Treseder, 2008). Possible explanations for this phenomenon include nitrogen application inhibiting the growth or ligninase activity of white rot fungi (Waldrop and Zak, 2006) or the production of brown, recalcitrant compounds induced by nitrogen application, leading to the accumulation of compounds toxic to microorganisms (Fog, 1988). This may also explain why the effect of NH₄^+^-N on microbial biomass after fertilization was insignificant. It is also possible that soil acidification subsequent to fertilization exerts an effect on microbial metabolic demand, which in turn leads to a decline in microbial biomass (Bardgett et al., 1999). While some studies indicate that nitrogen fertilizer increases microbial biomass, a few reports suggest a decrease in microbial biomass post-fertilization (Zheng et al., 2023). The differing ecosystem biomes and experimental durations may account for these inconsistencies (Jia et al., 2020).

However, in alpine meadows, fertilization under rest-grazing can promote the growth of nitrogen-rich plant species and increases aboveground vegetative litter, thereby enhancing microbial biomass (Zhou et al., 2023). This inconsistency may arise from differences in the fundamental conditions (soil texture, organic matter, moisture) and dominant species of the two grassland types (Li et al., 2016). The *Carex tibetikobresia* is the dominant species in swamping meadows, while *Carex parvula* predominates in alpine meadows (Zheng et al., 2000). The *Carex tibetikobresia* is a resource-harvesting plant characterized by a high specific root length, specific root area, and root nitrogen content, enabling rapid acquisition of water and nutrients (Laliberté, 2017). In contrast, *Carex parvula* is a resource-conservative plant with a low specific root length and high root tissue density, acquiring resources continuously with low metabolic intensity and long root life (Laliberté, 2017). Consequently, soil nutrients in the *Carex tibetikobresia* meadow are more abundant than in alpine meadows (Edwards et al., 2004), leading to a negative effect of fertilization treatment in the former. The implementation of fertilization and rest-grazing exhibited divergent impacts on plant community composition across distinct grassland ecosystems, a pattern corroborated by our experimental findings (Socher et al., 2013). Furthermore, research has demonstrated that the impact of rest-grazing on ecosystems is primarily influenced by the type of vegetation involved (Zhao et al., 2016a). Thus, the different responses of the two grasslands to rest-grazing and fertilization can be attributed to changes in vegetation type and effectiveness of soil properties.

### 4.2 The relationship between soil microbial community characteristics and potential drivers

Changes in soil microbial community composition occur along environmental gradients, primarily due to the characteristics of plants and soil (Dong et al., 2023). Therefore, we analyzed the correlation between environmental factors and microbial composition (Figures 7, 8). Our results indicate that, under rest-grazing measures, soil microbial community was significantly related to NH₄^+^-N, which may be attributed to several factors. First, NH₄^+^-N levels increased with longer rest-grazing duration, leading to a corresponding increase in the abundance of Act (Figure 4). This can be explained by the fact that rest-grazing promotes inorganic nitrogen availability by enhancing belowground primary productivity, thereby increasing organic matter input into the soil (Dong et al., 2020). NH₄^+^-N is incorporated in organic nitrogen, contributing to the long-term nutrient supply of the soil (Ren et al., 2024). Consequently, as inorganic nitrogen levels increase with rest-grazing, less NH_4_^+^-N is converted to nitrification, reducing nitrogen losses (Lv et al., 2024). This further suggests that rest-grazing enhances the retention of NH₄^+^-N. Second, NH₄^+^-N inhibited the growth of B and G^−^ bacteria (Figure 8), thereby altering the soil microbial community structure (LeBauer and Treseder, 2008). Additionally, NH₄^+^-N significantly influenced the supply level of organic matter, as indicated by the G^+^:G^−^ ratio (Figure 8). Recent research demonstrates that optimal nitrogen (N) inputs synergistically improve microbial community structure and functional dynamics via nutrient coupling effects, a finding that aligns with our experimental observations (Xue et al., 2025). This alteration in nutrient availability may impose stress on the microbial community, further contributing to changes in microbial composition (Chen et al., 2023).

Moreover, our research revealed that, the G^+^:G^−^ ratio was higher in the fertilization treatment than in the non-fertilization treatment (Figure 5). This suggests that soil microorganisms experience poor nutrient supply under fertilization, which may be detrimental to the sustainable functioning of the entire ecosystem (Lopes and Fernandes, 2020). A possible explanation for this observation is that the MBC content increased following fertilization, promoting an increase in the G^+^:G^−^ ratio (Figure 8). This finding is consistent with research indicating that increased nitrogen fertilizer enhances soil MBC (Bargali et al., 2018). This effect is primarily attributed to nitrogen fertilizer applications improving soil conditions, which allows microorganisms to utilize carbon sources more effectively (Tuo et al., 2023). Additionally, fertilizer application may increase soil enzyme activity, which has a significant positive correlation with MBC (Tuo et al., 2024). Recent research demonstrates that nitrogen fertilizer application alters soil biochemical processes, subsequently enhancing hydrolase enzymatic activity (Shi et al., 2025). Studies have demonstrated that urease, sucrase, and alkaline phosphatase activities positively correlate with MBC (Raiesi and Beheshti, 2015). Therefore, MBC is the predominant factor influencing microbial biomass following fertilization.

## 5 Conclusion

Our findings demonstrated that microbial communities were strongly associated with MBC and NH₄^+^-N. Compared to grazing, rest-grazing increased inorganic nitrogen levels, increasing Act and the ratio of gram-positive bacteria to gram-negative bacteria. Rest-grazing is an effective means of enhancing inorganic nitrogen availability in degraded grassland soils. Furthermore, without fertilization treatment, the ratio of fungi to bacteria and gram-positive bacteria to gram-negative bacteria reached a maximum at rest-grazing for 30 days. Fertilization mitigated the impact of NH₄^+^-N on microbial communities, with soil MBC emerging as a primary influencing factor. Fertilization reduced the biomass of total phospholipid fatty acids (PLFAs) and all PLFAs groups. Furthermore, microbial communities exhibited divergent responses to rest-grazing and fertilization between *Carex tibetikobresia* meadows and alpine meadows. This study assessed microbial community compositions, plant and soil properties, and their correlation with rest-grazing duration and fertilization. Given the above considerations, it is recommended that fertilization measures are not utilized on *Carex tibetikobresia* meadows, and that a 30-day rest-grazing duration is preferable. The results of the study highlight the importance of rest-grazing duration on microbial communities and provide a theoretical basis for grazing systems in degraded *Carex tibetikobresia* meadows.

## Data Availability

The original contributions presented in the study are included in the article/Supplementary material, further inquiries can be directed to the corresponding author.
