# Supplementary material for: Fertilization decreases the effect of ammonium nitrogen on microorganisms in Chinese Carex tibetikobresia meadows during rest-grazing
Source: Front Microbiol. 2025 Jun 18;16:1608011. doi: 10.3389/fmicb.2025.1608011 (PMC12213713; doi:10.3389/fmicb.2025.1608011)
Supplement: Supplementary file 1 [file Table_1.DOCX]

Table S1 The independent sample T-test between non-fertilization and fertilization

| Soil  Physicochemical  properties | *P*-value | | | | | | | | | |
| --- | --- | --- | --- | --- | --- | --- | --- | --- | --- | --- |
|  | SM | pH | NH_4_^+^-N | NO_3_^-^-N | TP | AP | TK | SOC | TN | Soil C: N |
| CK | 0.328 | 0.073 | 0.539 | 0.892 | 0.378 | 0.015* | 0.073 | 0.037 | 0.003** | 0.108 |
| 20d | 0.169 | 0.059 | 0.633 | 0.418 | 0.401 | 0.797 | 0.005** | 0.637 | 0.019* | 0.001** |
| 30d | 0.455 | 0.484 | 0.189 | 0.025* | 0.353 | 0.446 | 0.001** | 0.104 | 0.001** | 0.004** |
| 40d | 0.040* | 0.404 | 0.124 | 0.127 | 0.844 | 0.476 | 0.005** | 0.499 | 0.825 | 0.291 |
| 50d | 0.641 | 0.387 | 0.692 | 0.541 | 0.898 | 0.072 | 0.141 | 0.094 | 0.172 | 0.884 |

Note: * means significant difference between non-fertilized and fertilized under the same days of rest-grazing, *, P<0.05, **, *P*<0.01. CK: control check group; 20d: rest-grazing from June10th to June 30th; 30d: rest-grazing from May 30th to June 30th; 40d: rest-grazing from May 20th to June 30th; 50d: rest-grazing May 10th to June 30th; SM: soil moisture; pH: potential of hydrogen; NH_4_^+^-N: ammonium nitrogen; NO_3_^-^-N: nitrate nitrogen; TP: total phosphorus; AP: available phosphorus; TK: total potassium; SOC: soil organic carbon; TN: total nitrogen; Soil C: N: soil carbon-nitrogen ratio; (n=3).

Table S2 Redundancy analysis of soil microbial community and environmental variables using forward selection with a Monte Carlo permutation test

| Treatment | Variables | Explains  (%) | Contribution  (%) | F-ratio | P-Value | Axis | 1 | 2 |
| --- | --- | --- | --- | --- | --- | --- | --- | --- |
| Non- fertilization treatment | NH_4_^+^-N | 22.8 | 31.0 | 5.1 | 0.042 | Eigenvalues | 0.609 | 0.104 |
|  | D | 15.3 | 20.8 | 2.3 | 0.134 | Explained variation | 60.860 | 71.290 |
|  | SM | 12.7 | 17.3 | 2.1 | 0.160 | Pseudo-canonical correlation | 0.840 | 0.977 |
|  | TP | 8.7 | 11.9 | 2.2 | 0.164 | Explained fitted variation | 82.780 | 96.970 |
|  | J | 4.3 | 5.8 | 1.1 | 0.286 |  |  |  |
|  | TK | 3.5 | 4.7 | 0.9 | 0.380 |  |  |  |
| Fertilization treatment | MBC | 18.1 | 19.1 | 4.3 | 0.028 | Eigenvalues | 0.662 | 0.242 |
|  | MBN | 10.3 | 10.9 | 3.2 | 0.052 | Explained variation | 66.160 | 90.400 |
|  | SOC | 7.2 | 7.6 | 3.0 | 0.076 | Pseudo-canonical correlation | 0.982 | 0.990 |
|  | H | 13.2 | 13.9 | 2.1 | 0.142 | Explained fitted variation | 69.660 | 95.180 |
|  | MBC: MBN | 10.5 | 11.1 | 1.8 | 0.212 |  |  |  |
|  | J | 10.0 | 10.5 | 1.4 | 0.220 |  |  |  |

Note: NH_4_^+^−N: ammonium nitrogen; D: Simpson index; SM: soil moisture; TP: total phosphorus; J: Pielou index; TK: total potassium; MBC: soil microbial biomass carbon; MBN: soil microbial biomass nitrogen; SOC: soil organic carbon; H: Shannon-Weiner index; MBC: MBN: the ratio of soil microbial biomass carbon to soil microbial biomass nitrogen.
